# The Change in the Content of Nutrients in Diets Eliminating Products of Animal Origin in Comparison to a Regular Diet from the Area of Middle-Eastern Europe

**DOI:** 10.3390/nu12102986

**Published:** 2020-09-29

**Authors:** Kamila Kowalska, Jacek Brodowski, Kamila Pokorska-Niewiada, Małgorzata Szczuko

**Affiliations:** 1 Department of Human Nutrition and Metabolomics, Pomeranian Medical University in Szczecin, Broniewskiego 24, 71-460 Szczecin, Poland; mila1994kow@gmail.com; 2 Primary Care Department, Pomeranian Medical University in Szczecin, Żołnierska 48, 71-210 Szczecin, Poland; jacek.brodowski@pum.edu.pl; 3 Department of Toxicology, Dairy Technology and Food Storage, West Pomeranian University of Technology in Szczecin, Papieża Pawła VI 3, 71-459 Szczecin, Poland; kamila.pokorska@zut.edu.pl

**Keywords:** vegetarian diets, vegetarianism, vitamins, minerals, nutritional habits of Poles

## Abstract

Introduction: The diet of Poles became similar to the western style of nutrition. It is rich in saturated fats, it contains significant quantities of salt, and has very low fruit and vegetable content. On the other hand, introducing an incorrectly planned diet that eliminates animal products may be associated with the risk of deficiencies of certain vitamins and minerals. Taking into account the regular diet of Poles, a properly balanced vegetarian menu may be a better and safer choice for the proper functioning of the organism. Aim: The analysis of the content of individual types of vegetarian diets and a comparison with the menus of the regular diet of the Polish population. Materials and methods: 70 menus were subjected to a quantitative analysis, 10 menus for each 7 type of diet eliminating products of animal origin and regular diets without elimination. The caloricity of the designed diets was ±2000 kcal. The quantitative evaluation of the menus was performed using the Dieta 6d dietary program. Statistical significance was established at *p* ≤ 0.05. Results: It was observed that the regular diet of Poles (RD) featured the highest content of total fats, as well as saturated acids and cholesterol. The VEGAN diet was characterized by the lowest total protein content and the lack of wholesome protein and cholesterol. RD was characterized by the lowest average content of dietary fiber. The highest content of saccharose was observed in RD. Sodium content in RD significantly exceeded the recommended daily norm. RD featured insufficient content of the following minerals and vitamins: potassium, calcium, magnesium, iodine, Vitamin E, Vitamin C, folates, and Vitamin D. The norm for calcium has not been fulfilled also in milk-free and vegan diets. All of the analyzed diets lacked proper amounts of iodine and Vitamin D. The highest content of polyunsaturated fatty acids was observed in the VEGAN diet. The periodic elimination of meat and fatty dairy products should be included in the treatment of the metabolic syndrome, hypertensions, hyperlipidemia, obesity, and type 2 diabetes. Conclusions: The regular diet of Poles turned out to be more dangerous for health in terms of deficiencies than properly balanced diets eliminating products of animal origin.

## 1. Introduction

Humans are anatomically and physiologically fit to consume products both of plant and animal origin [1]. However, the current trends associated with mass production often result in people resigning from the consumption of meat and other products of animal origin. Other reasons for the elimination of such products from the diet include religious beliefs, views relating to health and ecology, as well as the economic situation [2]. Every type of diet that eliminates animal products should fulfill the need of an individual for nutrients, and in the case of their deficiencies, it is important to include supplementation. Properly balanced diets eliminating products of animal origin are not a threat to health. However, during the period of growth, pregnancy, or lactation, and in sportsmen, significant nutritional restrictions in the form of veganism may entail the risk of numerous nutritional deficiencies and illnesses (having in mind incorrectly planned diets). Despite the growing interest of people in vegetarian diets, the average consumption of meat in developed countries is still at a level that is too high, especially among men [3]. The current information on public health recommend the consumption of up to three portions of red meat (350g–500g per week), whereas processed meat products should be avoided or limited as much as possible [4]. Women more often become vegetarians than men [3]. Lower body mass index (BMI), cholesterol concentration, and blood pressure were observed in vegetarians, having a cardioprotective effect [5]. The risk of death caused by ischemic heart disease is 24% lower in vegetarians than in people who regularly consume meat. The conducted studies suggest that the vegetarian diet—eliminating red meat and poultry—and the lacto-vegetarian diet decrease the level of total cholesterol and low-density lipoprotein (LDL) cholesterol by about 10–15%, whereas the vegan diet—by about 15–25% [6]. Frequently occurring caloric restrictions in the use of vegetarian diets have an effect on life extension and protection against cancer [7]. Likewise, eliminating fried, smoked, or grilled foods, red meat, aflatoxin-contaminated foods, preserved salty meals, and alcohol [8]. Moreover, cancer risk is also reduced by introducing a diet rich in plant foods (e.g., vegetables, beans, fruit, and whole grains) and by reducing consumption of animal fat, meat, and fatty dairy products [9].

Vegetables and fruits are an important source of a wide range of bioactive ingredients and compounds, including antioxidants, vitamins (folic acid, carotenoids), glucosinolates, indoles, isothiocyanates, protease inhibitors, lycopene, phenolic compounds, and flavonoids that exhibit anti-cancer properties [10,11].

Studies conducted by the Homo Homini Opinion Institute in 2013 showed that the number of vegetarians in Poland is over a million, including 1.6% of lacto-vegetarians and 1.6% of vegans. A Centre for Public Opinion Research (CBOS) survey indicated that 26% of Poles followed some form of an elimination diet in 2014. The excluded products featured meat (11%), milk (9%), milk products (8%), fish (4%), and chicken eggs (4%) [12].

The aim of our study was to track the changes associated with the quantity of individual ingredients, including nutrients, fatty acids, amino acids, vitamins, and minerals. To achieve this, it was determined that all of the analyzed diets would include the same caloricity in accordance with the daily need of 2000 kcal.

## 2. Types of Diets Eliminating Products of Animal Origin, Which Were Analyzed in This Study

### 2.1. Semi-Vegetarian Diet

The elimination of red meat decreases the frequency of occurrence of type 2 diabetes and regulates carbohydrate metabolism [13,14]. Moreover, the risk of large intestine, breast, and prostate cancers was much higher in omnivores than in vegetarians [5]. Vegetarian diets are associated with increased consumption of vegetables and fruit rich in phytochemicals, dietary fiber, and antioxidants. These substances have a positive influence on health, e.g., they protect the organism against free radicals, simultaneously preventing the formation of neoplasms. Plant diets are also characterized by low concentrations of saturated fatty acids (SFA) and by high concentrations of polyunsaturated fatty acids (PUFA), having a positive influence on the lipid profile [15]. Soy often replaces meat in plant diets as it is a rich source of proteins and phytoestrogens, particularly isoflavones, which may play a protective role in reference to the development of breast cancer [16].

### 2.2. Lacto-Ovo-Vegetarian Diet

The lacto-ovo-vegetarian diet (LOV) is a type of vegetarianism that eliminates meat, meat products, and fish, but allows for the consumption of eggs and dairy products. People following this type of diet avoid products that simultaneously fulfill two criteria—“when this animal was alive, it had eyes and mommy”. Eggs in the diet are a necessary source of fatty acids, wholesome protein, choline, selenium, Vitamin A and B_12_ [17]. One of the constituents of yolk is cholesterol, which for many years has been considered the cause of increased risk of cardiovascular diseases (CVD). Therefore, it is recommended for omnivores to consume no more than 3 yolks per week. The most recent studies indicate that the presence of higher numbers of eggs in the weekly menu (more than 3) is not dangerous to health if other sources of cholesterol are reduced. Yolk is a valuable source of fatty acids, lecithin, choline, xanthophylls, immunoglobulin, and vitamins. Differences in the quantitative content of eggs may occur in the case of a different method of nutrition of hens, and as a result of special fodder additives [18].

### 2.3. Vegan Diet

The most restrictive type of vegetarianism is veganism, which eliminates all products of animal origin. Food products present in vegan menus include: cereal, fruit, vegetables, nuts, mushrooms, legumes, oils, and plant drinks. People choose the vegan diet mainly due to ethics (protection of animal rights), religious beliefs, and personal health. However, when applied for a longer period of time, this type of nutrition without proper balancing and supplementation results in negative health consequences. The main risk is the deficiency of vitamins and certain minerals that are necessary for the proper functioning of the human body [5]. It has been demonstrated that incorrectly balanced vegan diet may be the cause of the development of numerous neurological disorders, such as fear, depression, brainstorm, neuropathy, chronic tiredness, and insomnia [19]. Vegans also suffer from low concentrations and deficiencies of Vitamin B_12_, resulting from the exclusion of all products of animal origin, which are their only natural source [20]. B_12_ deficiencies may lead to megaloblastic anemia or demyelinating disease [21]. The vegan diet is characterized by high folate content, positively influencing the correct functioning of hematopoietic and nervous systems [15]. One of the factors that influences the development of CVD and neoplasms is obesity. It has been proven that vegans have lower average BMI than omnivores and other vegetarians, which decreases the risk of heart diseases and mortality resulting from ischemic heart disease [22]. The main sources of proteins in vegans include legumes, which include fiber and phytochemicals that help control glycemia, reducing the risk of developing type 2 diabetes [23].

### 2.4. Milk-Free Diet

The dairy-free or lactose-free diet is most often applied due to cow milk protein allergy (CMPA), lactose intolerance, lack of availability, and the increasingly popular trend. Products that are eliminated in the menu include milk and dairy products (cheese, yoghurt, cottage cheese, milk kefir, cream, buttermilk). Study results show that only 25% of the world’s population can breakdown lactose both in childhood and as adults. In most infants, the activity of intestinal lactase is the strongest in the perinatal period. However, after 2 years (from 2 to 12 years of age), two different groups appear, i.e., people with low and people with high lactase activity. Another case includes people that have the lactose breakdown ability throughout their entire life [24]. There are people that experience lactose intolerance, which is why after the consumption of milk and milk products they suffer from annoying symptoms referring to the gastrointestinal tract, as well as systemic symptoms. Colon microflora ferments the undigested lactose in the gut, leading to the creation of short-chain fatty acids (SCFA), hydrogen, carbon dioxide, and methane. Lactose intolerance symptoms can be untypical, e.g., headaches and dizziness, mouth sores, sore throat caused by the increase in the size of lymph nodes and muscles [25].

Dairy products play a key role in human diet as they are a rich source of vitamins and minerals, especially riboflavin, calcium, and wholesome protein [26,27]. However, dairy is characterized by low content of iron and folic acid [28]. It is recommended to consume lean dairy products due to the reduced content of saturated fatty acids (SFA), which are responsible for the increase of LDL cholesterol, and the increased risk of CVD. It has also been observed that the milk-free diet is helpful in the treatment of intestinal inflammations or acne inversa, which is associated with insulin metabolism [29].

### 2.5. Fish-Free Diet

Another type of the studied diet is one that eliminates fish. The consumption of fish and seafood has a positive influence on health—a fact that is supported by numerous studies. The main advantage of fish is the content of omega-3 fatty acids, Vitamin D and minerals, including iodine [30]. Some of the most important elements of the diet are fatty sea fish, i.e., salmon, herring, mackerel—the ones that are the richest in eicosapentaenoic acid (EPA) and docosahexaenoic acid (DHA) fatty acids. They influence such factors as the proper functioning of the circulatory system, and they are important for the proper development of the fetus, including the function of its neurons and retina, as well as for the immunity of the entire organism. EPA and DHA fatty acids are also associated with promising results in terms of the prevention and management of body mass and cognitive functions in people with very mild Alzheimer’s disease, a better prognosis after ischemic stroke in the treatment of illnesses with an inflammatory basis [31]. Vitamin D_3_ is naturally present in many products, but its richest sources include some fatty fish, oil extracted from fish liver, and caviar.

Numerous studies conducted in recent years have demonstrated that the increasing pollution of the environment has an influence on fish and shellfish. The increase in toxic substances originating from farms and the presence of heavy metals in the environment of water animals negatively influence human health via the food chain [32]. Fish feature increased concentrations of metals, such as arsenic, cadmium, chromium, mercury, and iron, which makes people avoid their consumption [33]. Other reasons for the limitation of the consumption of fish include fish and shellfish protein allergies, the specific smell which is unacceptable by some people, as well as high price, particularly of wild fish.

### 2.6. Regular Diet of Poles

In most cases, the regular diet of Poles (RD) includes all types of animal products, including red meat, white meat, eggs, milk, dairy, and—occasionally—fish. Recent studies have shown that the diet of Poles features excessive total fat content, including SFA and cholesterol. Insufficient consumption of PUFA and dietary fiber has also been observed [34]. Excessive amounts of salt and saccharose are frequently observed. All of these factors may be the cause of the increased risk of CVD the metabolic syndrome progressing together with diabetes and fatty liver. Other studies indicated that the menus of Poles are deficient in terms of folates, Vitamin D, thiamin, and niacin, and when it comes to minerals, they lack the proper concentrations of calcium, potassium, magnesium and iron in the group of women [35,36].

To conclude, our goal was to compare diets in terms of nutrient content, to determine any potential risk to health in the case of diets properly prepared by qualified dieticians, and to establish whether correctly balanced vegetarian diets could still pose a higher risk of nutritional deficiencies than the regular nutritional habits of Poles.

## 3. Materials and Methods

The study was conducted after receiving the permission of the Bioethics Committee of the Pomeranian Medical University (regulation no. KB-0012/116/15). The subjects gave written informed consent, and their confidentiality and anonymity were protected. The study population consisted of 30 people, including 18 men and 12 women. From this group, menus for the RD diet were selected from 10 people (equal number of sexes), corresponding to the assumed calorific value (2000 kcal). All subjects were Caucasian, potentially healthy with an average age of 39.26 ± 7.86 for women and 40.56 ± 6.43 for men.

### 3.1. Preparation of Menus

The study features the quantitative analysis of a total of 70 menus, 10 for each type of diet eliminating products of animal origin, as well as RD without elimination. Every menu has been prepared by qualified dieticians with the use of methods avoiding nutritional deficiencies, however, do not include supplementation. The caloricity of the developed menus was ±2000 kcal. The total energy from fat was 25–35%, proteins 10–17%, and carbohydrates complemented the rest of the caloricity. The supply of ingredients with water was not included in any of the menus.

Additionally, 30 menus were collected on the basis of a 24 h interview on nutrition conducted with adults, and the data was incorporated into a dietary program in order to evaluate caloricity and the content of individual nutrients. Out of the menus, those that fulfilled the inclusion criteria of ±2000 kcal were chosen. Then 29 ingredients were analyzed: energy, total protein, plant protein, animal protein, carbohydrates, dietary fiber, fat, cholesterol, omega-3 and omega-6 fatty acids, minerals: Na, K, Ca, P, Mg, Fe, Zn, Cu, Mn; vitamins soluble in water: C, B1, B2, B3, B6, B12; folic acid, vitamins soluble in fats: A, D, E; the participation of energy, fats and carbohydrates. The analysis also includes the concentration of exogenous and relatively exogenous amino acids.

### 3.2. Quantitative Analysis

The quantitative analysis of the menus was conducted using the Dieta 6 days dietary program, which is recommended by the National Institute of Food and Nutrition. The total caloricity of the menus has been divided into 3 main meals (breakfast, dinner, supper) and 2 additional meals (second breakfast and an evening meal fulfilling a complimentary role).

Types of the analyzed diets, prepared by dieticians:

- The basic diet (BD), which includes all product groups: meat, dairy, eggs, and fish;
- The modification of a vegetarian diet with fish (pescoveget-PV), which eliminates red and white meat, but allows for the consumption of eggs, dairy and fish;
- The milk-free diet (MFD), which eliminates all dairy products;
- The vegan diet (VEGAN), which eliminates all products of animal origin: meat, eggs, dairy, fish and honey;
- Diet that eliminates fish, seafood and shellfish (FFD);
- The lacto-ovo-vegetarian diet (LOV), which eliminates meat and fish, allowing for the consumption of eggs and dairy;
- The regular diet (RD); data collected on the basis of a 24 h nutrition interview of 10 Polish adults, with the selection of menus of 2000 kcal caloricity;

After creating a basic diet (BD) containing all protein sources from the diet, subsequent products were eliminated and replaced with substitutes. They were: lean dairy products (yoghurt, mozzarella, cottage cheese) and/or eggs and/or fish: cod, mackerel, salmon, tuna, herring, trout and/or vegetable milk, and legumes, tofu, seeds: almonds, nuts, cocoa, sesame, poppy seed, linseed, amaranth, pumpkin, sunflower seeds. The appropriate caloric value and the percentage of protein, fat, and carbohydrates in the diet were respected. The diets have been planned in order to be similar to the Polish diet with the best possible use of products available on the market that complemented possible shortages. The average content of nutrients was compared to the updated nutritional norms for the Polish population, developed by the National Institute of Food and Nutrition in 2017 [37]. Table 1 presents the current norms for nutrients developed by the Institute and determined at the level of the estimated average requirement (EAR). The table features ingredients that are present in Dieta 6d software.

### 3.3. Statistical Analysis

The statistical analysis was conducted using STATISTICA 13.3 (StatSoft, Cracow, Poland). The average ($\bar{x}$) and standard deviation (SD) were calculated for all individual nutrients in the analyzed diets. Analysis of variance (ANOVA) was used to determine the differences in the content of individual nutrients between the diets. Statistical significance was established at *p* ≤ 0.05. In accordance with the assumptions of the study, we observed no statistically significant differences in the caloricity of the analyzed diet types (*p* ≥ 0.05).

## 4. Results

### 4.1. Analysis of the Content of Proteins and Amino Acids

**Figure 1:**
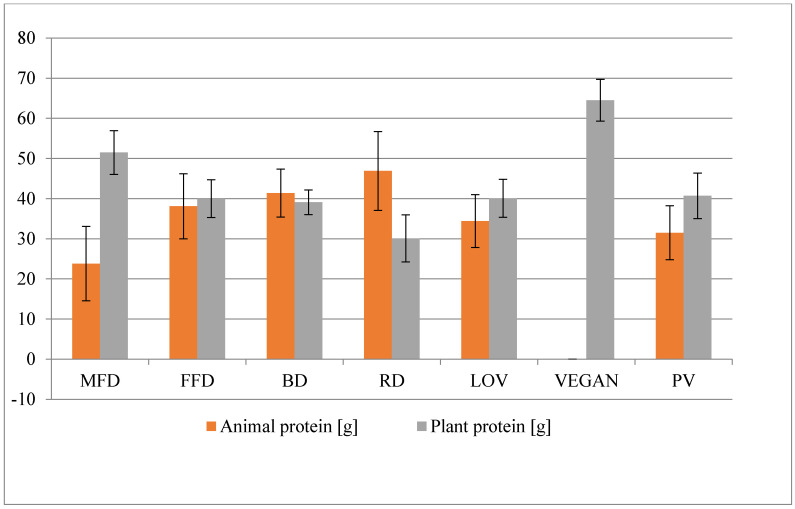
The average content of total plant and animal protein in the diets.

Statistically significant differences were observed in terms of plant and animal protein between MFD and the other diets (Figure 1). In the case of FFD, the content of animal protein in the menu significantly differs from RD, VEGAN, and PV, whereas for plant protein, significant differences are observed with reference to RD and VEGAN. The basic diet contains statistically significantly more animal protein than LOV, VEGAN, PV, and MFD (*p* < 0.05), but significantly lower content of plant protein than VEGAN and MFD. The content of plant protein in the regular diet is significantly lower than in the case of other diets. It has also been observed that there are statistically significant differences in the content of plant protein in the following pairs: LOV and VEGAN; VEGAN and PV (Table 2).

The lowest content of all exogenous amino acids was observed in the VEGAN diet (Table 3). Statistically significant differences were observed in the content of isoleucine, leucine, lysine, methioninee, phenylalanine, threonine, tryptophan, and valine between VEGAN and other diets. In terms of isoleucine, PV differed significantly from FFD and BD. MFD and BD showed a difference in the content of isoleucine, leucine, lysine, methionine, phenylalanine, and valine. In terms of leucine, significant differences were observed between BD and PV, and between MFD and LOV. FFD and MFD statistically significantly differed in the quantity of leucine, lysine, phenylalanine and valine. Statistical significance in terms of lysine content was observed for BD and RD, BD and LOV, BD and PV, FFD and PV. In terms of methioninee—MFD and RD, BD and PV, RD and PV. For threonine—BD and LOV, RD and LOV, PV and FFD, BD and RD. The content of tryptophan significantly differed between PV and FFD, BD. PV and BD were different in terms of valine content (Table 4).

### 4.2. Analysis of Fat Content

**Figure 2:**
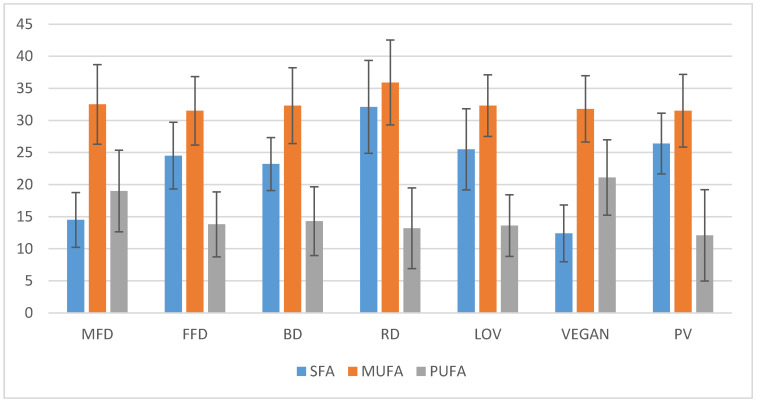
The content of saturated fatty acids (SFA), monounsaturated fatty acids (MUFA), and polyunsaturated fatty acids (PUFA) in diets (g/day).

The highest content of total fats, saturated fats, and monounsaturated fats was observed in the regular diet (RD). The VEGAN diet was characterized by the highest average content of PUFA (Figure 2). Statistically significant differences were observed in terms of fat content between RD and the other diets, between LOV and MFD, and between LOV and VEGAN (Figure 2, Table 5).

Statistically significant differences were observed in the content of long-chain PUFA between the following diets: FFD and MFD, FFD and BD, LOV and MFD, LOV and BD, VEGAN and MFD, VEGAN and BD, PV and MFD, and PV and BD (Table 5). The lowest content of long-chain PUFA was observed in VEGAN (Figure 2).

The average content of cholesterol was the highest in RD and exceeded the recommended daily intake, whereas VEGAN featured very little amounts of this constituent (Table 5). Statistically significant differences in the content of this constituent were observed between VEGAN and all other diets. In terms of cholesterol content, RD differed from MFD, FFD, BD, VEGAN, and PV; LOV differed in comparison to MFD, VEGAN, and PV, whereas PV was different when compared to RD, LOV, and VEGAN (Table 5).

### 4.3. Analysis of the Content of Carbohydrates

The highest content of carbohydrates (302.7 ± 22.17 g) was observed in VEGAN, the lowest (257.9 ± 24.29 g) in the RD. RD turned out to be deficient in terms of dietary fiber while other diets contained an average of over 35 g/day of this constituent: MFD—41 g, FFD—37 g, BD—37 g, LOV—37 g, and PV 38 g. Statistically significant differences were observed between RD and the other diets. VEGAN differed in terms of fiber content when compared to FFD, BD, RD, and LOV.

Statistically significant differences were observed in the content of saccharose between RD and the other diets. RD was characterized by the highest average content of this constituent and was at the level of 40 ± 13.73 g.

The lowest content of lactose was observed in MFD and VEGAN. No statistically significant differences were observed in the average content of starch in the analyzed diets (Table 6).

### 4.4. Analysis of Minerals

RD included the highest average content of sodium, which was a few times higher than the recommended dose. The lowest content of sodium was observed in VEGAN. Statistically significant differences were observed between RD and the other diets. In terms of sodium content, VEGAN differed from FFD, BD, RD, LOV, and PV (Table 7).

The regular diet (RD) did not fulfil the recommended dietary allowances (RDA) norm with reference to some minerals, such as potassium, magnesium, and iodine, and their average content was: potassium 2594.7 ± 594 mg, calcium 444.1 ± 157.7 mg, magnesium 261.1 ± 70.2 mg, and iodine 87.2 ± 45.0 µg. Insufficient level of calcium was also observed in MFD and VEGAN. FFD and BD contained incorrect quantities of calcium for pregnant women and children (according to the RDA norm). The highest average content of calcium was observed in LOV (1363.1 mg) The average requirement for phosphorus for the group (according to EAR) was fulfilled by all diets, but the lowest value was observed in RD (1099.6 mg), and the highest in FFD (1704.8 mg). The average content of phosphorus in RD does not fulfill the recommended daily norm for children, pregnant, and breastfeeding women (under 19 years of age). Magnesium content in RD did not meet the nutritional norm for men. The highest average content of iron was observed in VEGAN (19.8 mg), whereas the lowest in RD (11.2 mg). None of the analyzed diets fulfilled the recommended norm for iron for pregnant women. Women aged <50 should consume 18 mg of iron every day. Lower levels of this element were observed in FFD, BD, RD, LOV, and PV. RD also did not fulfill the norm for iron for 13–18 year-olds—boys and menstruating girls. The content of zinc was comparable in the analyzed diets, but the lowest content was observed in RD—10.6 mg. All diets featured lower than recommended content of zinc for breastfeeding women under 19. RD did not fulfill RDA for men, pregnant women, and breastfeeding women. The highest content of copper and manganese was observed in VEGAN (2.7 mg and 9.0 mg, respectively), while the lowest in RD (1.0 mg and 3.0 mg, respectively). RD featured lower than recommended content of copper for lactating women. All of the analyzed diets featured insufficient quantities of iodine when compared to nutritional norms (S1).

### 4.5. Vitamin Content Analysis

The lowest content of retinol was observed in VEGAN (22.7 µg), the highest in RD (682.7 µg). The most beta-carotene rich diet was BD (8942.3 µg), whereas RD included the lowest amounts of this constituent (1917.6 µg). RD did not meet the nutritional norm for Vitamin E in men, pregnant women and breastfeeding women. All of the analyzed diets fulfilled the norm for thiamin (Vitamin B_1_). RD and VEGAN featured insufficient amounts of riboflavin (Vitamin B_2_), which covers the needs of lactating women. MFD was the only diet that included the appropriate content of niacin (Vitamin B_3_). Insufficient average amount of this vitamin for pregnant women was observed in FFD, BD, RD, LOV, VEGAN, and PV, whereas in the case of breastfeeding women, deficiencies were observed in BD, RD, LOV, VEGAN, and PV. RD did not fulfill RDA in terms of Vitamin B_6_ content for pregnant women and lactating women. The lowest concentration of Vitamin C was observed in RD—70.9 mg. The recommended daily consumption of folates for pregnant women is 600 µg. All of the analyzed diets had lower values of this constituent. In the case of breastfeeding women, the recommended amount is 500 µg, but the norm was not fulfilled by MFD, FFD, BD, RD, and PV. VEGAN featured the lowest content of Vitamin B_12_ (2.2 µg), and this amount is not enough to cover the recommended daily consumption for men and women, including those that are pregnant or breastfeeding. FFD and PV were characterized by the lowest content of cobalamin with reference to lactating women. The highest content of this vitamin was observed in RD and MFD (4.1 µg). None of the analyzed diets fulfilled the norm for Vitamin D (Table 8 and Table S2).

## 5. Discussion

The nutritional habits of Poles deviate in many ways from the rules of rational nutrition. The diets are poor in vegetables, fruit, and wholegrain products, which results in the deficiency of some vitamins and minerals. Despite the same caloricity of the diets, there were differences between them in protein content. The source of proteins in a diet include products of animal and plant origin. The vegan (VEGAN) diet contained no products of animal origin and as such, it had the highest content of plant protein. Higher consumption of plant products is associated with the higher provision of individual constituents, such as fiber, potassium, and magnesium. Therefore, the decrease in the prevalence of CVD results from a number of factors, not just the consumption of plant protein [38]. The replacement of 1 standard portion of red meat (85 g) with three different plant sources of protein decreases the risk of coronary heart disease (CHD) by 13–30% according to the Nurses ‘Health Study [39], and by 7–19% in the combined control analyses Nurses ‘Health Study and Health Professionals Follow-Up [40]. The type of the consumed protein is important due to the various contribution of exogenous amino acids between plant and animal products [41]. Our study confirmed that the vegan diet and its derivatives pose a risk of insufficient supply of endogenous amino acids, relatively exogenous amino acids, as well as purely exogenous amino acids (leucine, isoleucine, lysine, methionine, threonine, phenylalanine, tryptophan, and valine). In the case of relatively exogenous amino acids, which include histidine, arginine, and serine, these amino acids can be produced in the body, but in exceptional situations, such as various illnesses, stressful events, or the period of quick growth, they should be supplied in appropriate amounts together with food [42]. In the context of the vegetarian diet, lysine is an important amino acid that requires special attention. In products of plant origin, its content is limited. To increase the supply of lysine, the vegetarian and (especially) vegan diets should be enriched with nuts and/or soy seeds. At the same time, appropriate supply of lysine has an influence on the decrease of the risk of heart diseases and some neoplasms as a result of the limitation of the activity of enzymes responsible for the lipogenesis and synthesis of cholesterol. Lysine also contributes to the reduction of the concentration of insulin-like growth factors (IGF) [43]. Another amino acid whose decreased supply is observed in the vegetarian diet is methionine. During the course of various transformations, methionine is transformed into taurine and homocysteine. For many people following the vegetarian diet, this amino acid reduces the assimilation of other amino acids [44]. When it comes to mental health, tryptophan is important. This amino acid is necessary for the production of serotonin, which is responsible for feeling well, the regulation of sleep, and it also prevents hyperactivity in children. Moreover, it is transformed into melatonin, and it also influences the secretion of hormones that support the synthesis of pyridoxine and niacin. The richest source of tryptophan is turkey meat, milk, and dairy. Because most vegetarian diets exclude these products, tryptophan supply is reduced in these diets, and tryptophan is also a limiting amino acid in our menus. It is used in the synthesis of neurotransmitters and, as such, its significant deficiency causes a specific depressive reaction [45,46]. There are ways of increasing the biological quality of the consumed plant protein through the organization of menus in a way that would supplement the content of protein products with the missing amino acids. One of the ways is to combine legume products with grain seeds in one meal, e.g., beans with rice. In comparison to animal proteins, plant proteins have lower contents of leucine, lysine, methionine, and tryptophan [42].

Vegetarian diets are associated with the lower level of cholesterol in the plasma and lower blood pressure. However, this is strongly associated with the lifestyle of vegetarians—these people are usually non-smokers, they do not drink alcohol, and are physically active.

The regular diet of Poles was characterized by the highest percentage of fat, unsaturated fatty acids, and cholesterol in comparison to other menus. This correlates with the increased risk of developing ischemic heart disease, atherosclerosis, and cancers—prostate, breast, or colon. Circulatory system diseases have been the main cause of death in the Polish population in recent decades [43]. The lowest content of PUFA was observed in FFD, RD, and PV. PUFA have a positive influence on the functioning of the circulatory system, e.g., they contribute to the decrease in the level of cholesterol, they lower arterial blood pressure, prevent the development of clots, and increase the strength of heart contraction [47].

High content of red meat, processed meat products, and eggs in these menus was the reason for the excessive consumption of cholesterol and saturated fatty acids, which are a significant factor contributing to deaths resulting from CVD. High level of cholesterol is also associated with the development of colorectal cancer [48]. Excessive levels of cholesterol also contribute to the development and progression of neurodegenerative diseases, such as Alzheimer’s disease and Parkinson’s disease [49]. Cholesterol is necessary for the proper development of the fetus in the first stages of pregnancy. After being born, about 40-50% of the child’s cholesterol intake comes from mother’s milk. Because of that, the VEGAN diet should not be recommended to pregnant or breastfeeding women [50].

Assimilable carbohydrates that are digested in the gastrointestinal tract are responsible for the supply of energy to muscles, the brain, heart, intestines, and erythrocytes. Dietary fiber (cellulose, hemicellulose, pectin, lignin) is not digested by enzymes in the gastrointestinal tract. The main functions of dietary fiber in the body are: the reduction of the level of cholesterol, glucose and insulin, the stimulation of fermentation processes in the intestine, the decrease in the time of intestinal passage, and the increase in the volume of stool [37]. According to WHO/FAO, the daily consumption of 25 g of fiber enables the correct functioning of the body. Basing on our original study, the lowest and insufficient content of fiber was observed in the RD. The insufficient supply of fiber in the diet may be associated with the development of disorders in the functioning of intestines, as well as with the increase in the risk of coronary artery disease and type 2 diabetes [51]. The main products of fermentation bacteria in the intestines are SCFA, especially acetate, propionate, and butyrate. They have many properties that are beneficial to health, they are responsible for feeling full, and they stimulate the immune system. In the case when there is a deficiency of dietary fiber, microbes shift to less favorable energy sources [52]. Moreover, prolonged consumption of high-fat and high-saccharose diet may lead to the death of the positive species of gut microflora [53]. Despite the numerous health benefits related to the high consumption of fiber, its excessive intake can have negative consequences. Products rich in food fiber, i.e., legumes, nuts, tofu, and some cereals are characterized by high content of phytic acid. Phytates may bind with some minerals, e.g., iron, zinc, and calcium, forming insoluble complexes, reducing their assimilation in the digestive tract [54]. This is why when using diets based on plant products (mainly legumes and cereals as in VEGAN and PV) it is important to control the levels of minerals in the body.

The regular diet was characterized by the highest content of saccharose out of all of the analyzed diets. This was caused by the presence of significant amounts of candy and sugar in the diet, the latter being added, e.g., to coffee, tea, and processed foods. Saccharose, which consists of glucose and fructose, also occurs naturally in honey, fruit, and vegetables, but in significantly lower quantities than in ready-made products prepared by the food industry. Excessive consumption of sugar is associated with many negative health aspects, such as circulatory system diseases, obesity, type 2 diabetes, caries, cirrhosis, and dementia [55]. Saccharose in the rest of the analyzed diets mainly originates from fruit, vegetables, and honey which, apart from being a source of sugar, include numerous valuable vitamins, minerals, as well as fiber.

The highest average content of lactose was observed in LOV, PV, and FFD. Very small amounts of milk sugar were observed in MFD and VEGAN, which originated from bread added in the dietetics program, though there should be no trace of this sugar in the menus at all. MFD and VEGAN could be appropriate for people with confirmed lactose intolerance and the incorrect assimilation of lactose in the digestive tract. Studies show that the risk of symptoms after the consumption of lactose depends on the dose of lactose, lactose expression, intestinal flora, and the sensitivity of the digestive tract [56]. When treating lactose intolerance, it is recommended to reduce lactose consumption, not eliminate it from the diet completely because in blind studies, most patients that reported the intolerance tolerated at least 12 g of lactose (which is equivalent to 250 mL of milk), and up to 18 g with the consumed foods [57]. A study conducted by Staudacher et al. regarding a diet poor in fermentable oligosaccharides, disaccharides, monosaccharides and polyols (FODMAP) indicated the improvement of symptoms in 86% of patients with the irritable bowel syndrome (IBS), in comparison to 49% in the case of a standard dietary intervention [58]. FODMAP is a diet that includes low contents of fermenting oligo-, di- and monosaccharides, as well as polyols, so fructose, lactose, fructans, galactans, and artificial sweeteners like sorbitol, mannitol, maltitol, and xylitol. All of these constituents are poorly absorbed in the small intestine, they are osmotically active (they can have laxative effects, they influence intestinal motility), and they are quickly fermented by intestinal bacteria. FODMAP aims at reducing or eliminating the presence of such symptoms as flatulence, stomachache, nausea, diarrhea, and constipation [59].

The regular diet of Poles was characterized by significantly higher consumption of sodium. Only VEGAN menus were characterized by lower levels of this constituent. High-sodium diet significantly increases the risk of developing hypertension, insulin resistance, dyslipidemia, and hipoadiponectemia [60]. The widespread supply of sodium is considered as one of the main causes of death resulting from circulatory system diseases. Sportsmen require higher intake of sodium, especially those that exercise in high temperatures. Higher sodium intake is also important for patients with insufficiency of the adrenal cortex and thyroid. During intense physical activity, contestants lose this element along with sweat [61]. Because of that, VEGAN may not be the right choice for people that do intense exercise.

Out of all of the analyzed diets, only RD was characterized by insufficient levels of potassium, when compared to the valid norm. Incorrect supply of this element may be associated with the increased risk of stroke and other circulatory system diseases [62]. Studies conducted by Zhang et al. indicate that excessive consumption of sodium positively correlated with increased systolic blood pressure and hypertension, and that the consumption of potassium negatively correlate with both of these disorders. Furthermore, the ratio of sodium and potassium was also important in the prevention of these problems [63].

The analysis of diets in terms of calcium content revealed that MFD, RD, and VEGAN contain insufficient amounts of this element. Well-absorbed sources of calcium are milk and milk products. Other sources include small fish (consumed with bones), beans, kale, parsley leaves, nuts, almonds, sesame seeds, and poppy seeds. It has to be highlighted that calcium originating from plant sources is less efficiently absorbed than calcium from milk and its products, which is associated with, e.g., the presence of lactose, which amplifies the absorption of this element [64]. However, it is emphasized that the best absorbed sources of calcium are vegetable with low in oxalate [65]. MFD completely eliminated milk products, this is why the average content of this macroelement was so low despite the use of other products that are its source. Due to the fact that the consumption of minerals with water was not taken into account, deficiencies in all types of diets, especially in terms of calcium, may be much smaller. The right supplementation of calcium and Vitamin D is of key importance for the prevention of the progressing loss of bone mass. In the case of postmenopausal women, it is recommended to consume a daily total of 1200 mg of calcium originating from food and supplements, and to supplement the diet with 800–2000 IU of Vitamin D. The supplementation is insufficient to prevent bone breaking in persons with osteoporosis. However, this is an important addition to a pharmacological intervention [66].

Phosphorus deficiencies were not observed in the studied diets. However, the proportion of Ca:P should be 1:1 to maintain the proper state of the skeleton. Mineral metabolism dysfunctions are the frequent complications of chronic kidney disease (CKD). A damaged kidney is not able to fully dispose of a phosphorus charge, leading to, e.g., secondary hyperparathyroidism. Studies conducted by Moe et al. indicated that protein products rich in phosphorus, i.e., cereals and legumes, are a better source of protein for people with CKD. The results of this study show that the use of the vegetarian diet in patients with CKD leads to the reduction of the level of phosphorus in the serum, when compared to a diet that includes meat [67].

The analyzed RD did not fulfill the RDA norm for magnesium with reference to men. Studies conducted by Adebamowo et al. indicated that a magnesium-, potassium-, and calcium-rich diet may contribute to the decrease in the risk of stroke in men [68]. Generally speaking, no pathological states associated with low magnesium consumption have been observed, but a small to moderate deficiency of this element resulting from chronic stress may significantly contribute to the presence of such illnesses as atherosclerosis, hypertension, osteoporosis, diabetes, and cancer [69]. Coffee, which is often consumed in large amounts by the Polish population, is a factor that is commonly believed to decrease the assimilation of magnesium, which favors many pathologies [70,71].

Iron present in food products has many forms and is usually classified as heme and nonheme iron. All of the analyzed diets fulfilled RDA for iron, but they differed in terms of its origin. In the case of meat-eliminating diets, it is mainly nonheme iron, which can occur in products in the form of various complexes, which may improve or weaken its absorption. An example of substances that significantly reduce the absorption of iron in the digestive tract are phytates and tannins of plant origin [72]. This is why the content of nonheme iron in the diet should be several times higher than heme iron.

RDA norms for such minerals as zinc, copper, and manganese have been fulfilled by all of the analyzed diets. However, taking into account factors that interrupt absorption and assimilation in the digestive tract, the supply of these constituents may turn out to be too low. A frequent factor facilitating this process is animal protein, which is not present in VEGAN. It has been demonstrated that the supplementation with zinc has a protective effect on the epithelial barrier of intestines and helps in various pathologies, including chronic alcohol consumption, oxidative stress, diarrhea, chronic fatigue syndrome, colitis, other gastrointestinal problems, and even some neurological disorders. However, zinc deficiency may result from the wide use of proton-pump inhibitor medicines, diets including large amounts of products rich in phytates and the decreasing consumption of meat and fish [50,73].

The main sources of copper are food products (75%) and drinking water (25%). Genetic illnesses connected to the disturbed metabolism of this element include Menkes disease, associated with bad absorption, and Wilson’s disease, in which the excretion of iron is disturbed. Infants are more vulnerable to the deficiency than adults. This is true especially for premature infants because the fetus absorbs copper in the last months of pregnancy. Children that do not go through breastfeeding require supplementation in the first year of their lives [74]. However, high copper consumption with trans-fat and saturated fatty acids has been associated with the accelerated decrease in cognitive functions of the elderly [75].

Manganese is a necessary element that is required for the proper functioning of the immune system, the regulation of the level of sugar in the blood, cellular energy, reproduction, digestion, bone growth, blood coagulation, hemostasis, and protection against reactive oxygen species. Manganese deficiencies are rarely observed because it is available in many food products. The absorption of manganese is strictly regulated in intestines and no toxicity resulting from its excessive intake with the diet was observed. The toxicity of manganese in the world results from environmental pollution, including the pollution of air and drinking water [76].

All of the analyzed diets were characterized by insufficient iodine content when compared to the norm. However, the addition of salt was not included in the meals. Thanks to fortification (in Poland in the form of potassium iodide), salt is the main source of iodine in the diet of Poles. The main sources of this constituent include sea fish, which were not present in FFD, LOV, VEGAN, and PV. This is why the average content of iodine in these diets is the lowest. The main results of the deficiency are goiter and hypothyroidism. In pregnant women, insufficient consumption of iodine may be associated with impaired psychomotor development of children, the risk of miscarriage or endemic cretinism. The impaired mental and somatic development may result from the deficiency of iodine in children and teenagers [77].

All of the analyzed diets fulfilled the norm for Vitamin A, sometimes significantly exceeding the recommended values. The lowest content of retinol was observed in VEGAN because the diet completely eliminates products of animal origin, which are its source. RD has the lowest average content of beta-carotene; a carotenoid present in plant products. The reason for the limited assimilation of Vitamin A may be the excessive intake of fiber or alcohol, excess amounts of iron, nitrates, nitrites, and free radicals in the body, as well as insufficient levels of zinc [41]. The symptoms of Vitamin A deficiency include weaker sight, skin dryness, the weakening of mucous membranes, higher vulnerability to infections.

The diets were properly balanced in terms of Vitamin E, only RD did not fulfill the RDA norm for men. Vitamin E also influences the efficiency of muscles and the production of sperm. Therefore, its appropriate supply is very important in men [78].

The effects of vitamins from the B family were observed in many aspects, including brain function, energy production, DNA and RNA synthesis and repair, as well as in the synthesis of numerous neurochemical substances and signaling particles. Insufficient amounts of B-family vitamins are associated with inflammatory processes and oxidative stress as indicated by the increased concentration of homocysteine in blood plasma [79]. The average content of B-family vitamins in the analyzed diets fulfilled the norm of the recommended consumption for the Polish population. The source of these vitamins includes both products of plant and animal origin. The only exception is Vitamin B_12_, which can be found only in animal foods. The VEGAN diet achieved the norm for this Vitamin Because the products used in the menus, i.e., soy milk or tofu, were enriched. The group of B vitamins includes folates (Vitamin B_9_). The regular diet of Poles turned out to be insufficient in terms of this nutrient. The methylenetetrahydrofolate reductase (MTHFR) 3 gene codes the methylene tetrahydrofolate reductase enzyme, which participates in the metabolism of folates, homocysteine, and methionine. MTHFR transforms folic acid from food into an active form, which can be used by the organism. This way, MTHFR influences the transformation of toxic homocysteine into methionine with the participation of folic acid. The presence of the C677T mutation of the MTHFR gene leads to the deficiency of folic acid and the accumulation of homocysteine. It is estimated that about 15% of the Polish population has the mutation of the MTHFR gene. More efficiently assimilated vitamins are those that originate from the diet, not supplements. The most valuable sources of B-family vitamins are meat, fish, seafood, nuts, liver, green leafy vegetables, yeast, and eggs. People with the MTHFR gene mutation should be supplemented with methylated folic acid because this is the only form that will be assimilated by the body. The C677T polymorphism of methylene tetrahydrofolate reductase is associated with various illnesses, i.e., circulatory system diseases, neoplasms, neurological diseases, diabetes, or psoriasis [80].

Vitamin C (ascorbic acid) has strong antioxidant properties, is well assimilated from the digestive tract, and its excess is removed with urine. The main sources of ascorbic acid in a diet are fruit and vegetables, but there are huge losses of this constituent during heat treatment and storage, even >75% when compared to fresh, raw product [41]. The regular diet (RD) was characterized by the lowest content of Vitamin C and, at the same time, it did not fulfill the norms of consumption. The remaining diets (MFD, FFD, BD, LOV, VEGAN, and PV) covered the norm for this vitamin. The symptoms of scurvy or Vitamin C deficiency include swelling of the lower limbs, bleeding of gums, tiredness, and hemorrhages, as well as psychological issues, including depression, hysteria, and social introversion [81].

Vitamin D deficiency is the main problem of public health in the entire world in all age groups, even in countries where it is generally assumed that UV radiation is sufficient to prevent this deficiency or in industrialized countries where fortification has been conducted for years.

The causes of Vitamin D deficiency:

- the use of sunscreen,
- elderly age,
- obesity,
- malabsorption,
- kidney and liver diseases,
- use of anticonvulsants.

Due to the fact that it is very difficult to supplement proper amounts of Vitamin D with food, none of the analyzed diets fulfilled the norm for this constituent. Regardless of whether the diet eliminated products of animal origin or not, supplementation is necessary, particularly in the autumn-winter period in the temperate climate that is present in Poland. It has been demonstrated that Vitamin D stimulates the absorption of calcium in the intestines [82]. Vitamin D deficiency is usually manifested through the deformation of bones (rickets) or hypocalcaemia in infancy and childhood, as well as through pain and musculoskeletal weakness in adults. Many other health problems, including circulatory system diseases, type 2 diabetes, several neoplasms, and autoimmune diseases, can be associated with Vitamin D deficiency [83].

The analysis of menus prepared by qualified dieticians will make it possible to avoid some deficiencies. However, it is important to take into account the fact that most people who follow this type of diet do it rather poorly, which—in most cases—leads to even higher deficiencies, including calorie deficiencies, especially in the VEGE diet. An important assumption of this study was the same caloric value for every type of the analyzed diets.

Moreover, dietary fiber plays a key role in many metabolic processes not only directly related to the function of the intestine. Vegetable fiber is used by the intestinal microbes (stimulating growth of intestinal microbes) to synthesize SCFA, which support healthy colonic epithelial cells [84]. Fiber consumption directly affects stool bulkiness, fecal pH, and intestinal transit time. The end products (acetate, propionate, and butyrate) produced by microorganisms affect enhancing various blood parameters (glucose, insulin) and the manner of bowel movements. The study showed that the numbers of bifidobacteria, lactobacilli, and methanogens were significantly decreased in the colon of patients with mixed refractory constipation [84,85]. Taking into account the above considerations, conclusions were drawn. Periodic implementation of vegetarian diets may help in the treatment of several diseases and symptoms associated with the metabolic syndrome, such as hypertension, hyperlipidemia, obesity, type 2 diabetes, and cardiovascular diseases. The implementation of diets that eliminate products of animal origin can be risky for pregnant or breastfeeding women, children, and the elderly.

## 6. Conclusions

The regular diet of Poles featured the highest total content of fats and the highest content of saturated fatty acids and cholesterol. The regular diet of Poles, at 2000 kcal caloricity, turned out to be more hazardous to health in terms of deficiencies than properly balanced diets with the same caloricity that eliminated products of animal origin. Considering assimilation capabilities, metabolism and the source of vitamins and minerals in individual menus, it was impossible to clearly determine a better way of fulfilling the requirements for these constituents in vegetarian diets. Diets that eliminated products of animal origin often require additional supplementation and the constant monitoring of mineral and vitamin levels in blood plasma.

It is important to pay attention to the nutritional education of Poles, both with reference to those that implement diets featuring all product groups, as well as those that follow vegetarian diets.

## Figures and Tables

**Table 1:** Nutrient norms for men and women over 19 years of age, determined on the basis of the EAR.

Nutrients	Women	Men
Vitamin A (µg)	500	630
Vitamin D (µg)	15	15
Vitamin E (mg)	8	10
Vitamin C (mg)	60	75
Vitamin B_1_ (thiamin) (mg)	0.9	1.1
Vitamin B_2_ (riboflavin) (mg)	0.9	1.1
Vitamin B_3_ (niacin) (mg)	11	12
Vitamin B_6_ (mg)	1.1	1.1
Folates (µg)	320	320
Vitamin B_12_ (cobalamin) (µg)	2.0	2.0
Calcium (mg)	800	800
Phosphorus (mg)	580	580
Magnesium (mg)	255	330
Iron (mg)	8	6
Zinc (mg)	6.8	9.4
Copper (mg)	0.7	0.7
Iodine (µg)	95	95
Manganese (mg)	1.8	2.3
Sodium (mg)	1500	1500
Potassium (mg)	3500	3500

EAR, estimated average requirement.

**Table 2:** The average content of proteins between the diets (g/day).

Total Protein							
	MFD	FFD	BD	RD	LOV	VEGAN	PV
$\bar{x}$ ± SD (g/day)	76.3 ± 4.13	79.2 ± 6.18	81.5 ± 4.38	77 ± 10.04	75.5 ± 5.76	66.1 ± 4.52	73.2 ± 7.94
MFD	-	0.3196	0.0770	0.8023	0.7777	**0.0008**	0.2904
FFD	0.3196	-	0.4298	0.4550	0.2029	**<0.0001**	0.0426
BD	0.0770	0.4298	-	0.1270	**0.0415**	**<0.0001**	**0.0057**
RD	0.8023	0.4550	0.1270	-	0.5946	**0.0003**	**0.1924**
LOV	0.7777	0.2029	**0.0415**	0.5946	-	**0.0018**	0.4368
VEGAN	**0.0008**	**<0.0001**	**<0.0001**	**0.0003**	**0.0018**	-	**0.0162**
PV	0.2904	0.0426	**0.0057**	**0.1924**	0.4368	**0.0162**	-

$\bar{x}–\mathrm{average}\;\mathrm{value};\;$SD—standard deviation; bold—statistically significant differences (*p* ≤ 0.05); MFD, milk-free diet; FFD, diet that eliminates fish, seafood and shellfish; BD, basic diet; RD, regular diet; LOV, lacto-ovo-vegetarian diet; PV, pascoveget.

**Table 3:** The average content of exogenous amino acids in the analyzed diets (mg/day).

Amino Acid (mg)	$\mathrm{MFD}\;\bar{x}\;\pm \;\mathrm{SD}$	$\mathrm{FFD}\;\bar{x}\;\pm \;\mathrm{SD}$	$\mathrm{BD}\;\bar{x}\;\pm \;\mathrm{SD}$	$\mathrm{RD}\;\bar{x}\;\pm \;\mathrm{SD}$	$\mathrm{LOV}\;\bar{x}\;\pm \;\mathrm{SD}$	$\mathrm{VEGAN}\;\bar{x}\;\pm \;\mathrm{SD}$	$\mathrm{PV}\;\bar{x}\;\pm \;\mathrm{SD}$
Isoleucine	3413 ± 375	3703 ± 444	3829 ± 352	3654 ± 492	3528 ± 282	2737 ± 210	3360 ± 410
Leucine	5428 ± 536	6145 ± 621	6298 ± 553	5815 ± 843	5970 ± 533	4570 ± 407	5748 ± 662
Lysine	4180 ± 557	5008 ± 735	5317 ± 506	4710 ± 740	4518 ± 486	3121 ± 442	4397 ± 646
Methionine	1543 ± 298	1721 ± 249	1822 ± 231	1815 ± 256	1642 ± 197	940 ± 88.3	1530 ± 213
Phenyl-alanine	2962 ± 250	3602 ± 314	3649 ± 293	3520 ± 502	3558 ± 291	2686 ± 301	3421 ± 355
Threonine	2931 ± 85	3043 ± 380	3198 ± 302	2147 ± 434	2852 ± 248	2376 ± 220	2739 ± 328
Tryptophan	972 ± 118	1030 ± 118	1050 ± 107	965 ± 142	957 ± 91.3	810 ± 69	926 ± 126
Valine	4020 ± 426	4532 ± 459	4640 ± 427	4263 ± 585	4396 ± 366	3267 ± 242	4234 ± 477

$\bar{x}–\mathrm{average}\;\mathrm{value};\;$SD—standard deviation; bold—statistically significant differences (*p* ≤ 0.05).

**Table 4:** The differences in the content of exogenous amino acids.

Isoleucine	MFD	FFD	BD	RD	LOV	VEGAN	PV
MFD	-	0.0906	**0.0164**	0.1585	0.4969	**0.0002**	0.7561
FFD	0.0906	-	0.4583	0.7714	0.3044	**<0.0001**	**0.0465**
BD	**0.0164**	0.4583	-	0.3033	0.0796	**<0.0001**	**0.0072**
RD	0.1585	0.7714	0.3033	-	0.4599	**<0.0001**	0.0869
LOV	0.4969	0.3044	0.0796	0.4599	-	**<0.0001**	0.3234
VEGAN	**0.0002**	**<0.0001**	**<0.0001**	**<0.0001**	**<0.0001**	**-**	**0.0005**
PV	0.7561	**0.0465**	**0.0072**	0.0869	0.3234	0.0005	-
**LEUCINE**	MFD	FFD	BD	RD	LOV	VEGAN	PV
MFD	-	**0.0104**	**0.0021**	0.1587	**0.0500**	**0.0024**	0.2434
FFD	**0.0104**	-	0.5744	0.2285	0.5219	**<0.0001**	0.1479
BD	**0.0021**	0.5744	-	0.0798	0.2314	**<0.0001**	**0.0466**
RD	0.1587	0.2285	0.0798	-	0.5693	**<0.0001**	0.8044
LOV	**0.0500**	0.5219	0.2314	0.5693	-	**<0.0001**	0.4148
VEGAN	**0.0024**	**<0.0001**	**<0.0001**	**<0.0001**	**<0.0001**	**-**	**<0.0001**
PV	0.2434	0.1479	**0.0466**	0.8044	0.4148	**<0.0001**	-
**LYSINE**	MFD	FFD	BD	RD	LOV	VEGAN	PV
MFD	-	**0.0029**	**<0.0001**	0.0517	0.2111	**0.0002**	0.4193
FFD	**0.0029**	-	0.2513	0.2708	0.0718	**<0.0001**	**0.0259**
BD	**<0.0001**	0.2513	-	**0.0267**	**0.0040**	**<0.0001**	**0.0010**
RD	0.0517	0.2708	**0.0267**	-	0.4141	**<0.0001**	0.2462
LOV	0.2111	0.0718	**0.0040**	0.4141	-	**<0.0001**	0.6540
VEGAN	**0.0002**	**<0.0001**	**<0.0001**	**<0.0001**	**<0.0001**	**-**	**<0.0001**
PV	0.4193	**0.0259**	**0.0010**	0.2462	0.6540	**<0.0001**	-
**METHIONINE**	MFD	FFD	BD	RD	LOV	VEGAN	PV
MFD	-	0.0860	**0.0079**	**0.0097**	0.3362	**<0.0001**	0.8967
FFD	0.0860	-	0.3222	0.3604	0.4411	**<0.0001**	0.0655
BD	**0.0079**	0.3222	-	0.9393	0.0810	**<0.0001**	**0.0055**
RD	**0.0097**	0.3604	0.9393	-	0.0947	**<0.0001**	**0.0068**
LOV	0.3362	0.4411	0.0810	0.0947	-	**<0.0001**	0.2758
VEGAN	**<0.0001**	**<0.0001**	**<0.0001**	**<0.0001**	**<0.0001**	**-**	**<0.0001**
PV	0.8967	0.0655	**0.0055**	**0.0068**	0.2758	**<0.0001**	-
**PHENYLALANINE**	MFD	FFD	BD	RD	LOV	VEGAN	PV
MFD	-	**<0.0001**	**<0.0001**	**0.0005**	**0.0002**	0.0729	**0.0035**
FFD	**<0.0001**	-	0.7588	0.5861	0.7736	**<0.0001**	0.2358
BD	**<0.0001**	0.7588	-	0.3954	0.5524	**<0.0001**	0.1372
RD	**0.0005**	0.5861	0.3954	-	0.7970	**<0.0001**	0.5183
LOV	**0.0002**	0.7736	0.5524	0.7970	-	**<0.0001**	0.3673
VEGAN	0.0729	**<0.0001**	**<0.0001**	**<0.0001**	**<0.0001**	**-**	**<0.0001**
PV	**0.0035**	0.2358	0.1372	0.5183	0.3673	**<0.0001**	-
**THREONINE**	MFD	FFD	BD	RD	LOV	VEGAN	PV
MFD	-	0.4410	0.0679	0.1386	0.5852	**0.0003**	0.1860
FFD	0.4410	-	0.2833	0.4715	0.1902	**<0.0001**	**0.0386**
BD	0.0679	0.2833	-	0.7217	**0.0191**	**<0.0001**	**0.0022**
RD	0.1386	0.4715	0.7217	-	**0.0447**	**<0.0001**	**0.0061**
LOV	0.5852	0.1902	**0.0191**	**0.0447**	-	**0.0015**	0.4334
VEGAN	**0.0003**	**<0.0001**	**<0.0001**	**<0.0001**	**0.0015**	**-**	**0.0140**
PV	0.1860	**0.0386**	**0.0022**	**0.0061**	0.4334	**0.0140**	-
**TRYPTOPHAN**	MFD	FFD	BD	RD	LOV	VEGAN	PV
MFD	-	0.2565	0.1301	0.8809	0.7632	**0.0020**	0.3587
FFD	0.2565	-	0.6988	0.1998	0.1527	**<0.0001**	**0.0426**
BD	0.1301	0.6988	-	0.0971	0.0710	**<0.0001**	**0.0167**
RD	0.8809	0.1998	0.0971	-	0.8796	**0.0031**	0.4417
LOV	0.7632	0.1527	0.0710	0.8796	-	**0.0048**	0.5362
VEGAN	**0.0020**	**<0.0001**	**<0.0001**	**0.0031**	**0.0048**	**-**	**0.0245**
PV	0.3587	**0.0426**	**0.0167**	0.4417	0.5362	**0.0245**	-
**VALINE**	MFD	FFD	BD	RD	LOV	VEGAN	PV
MFD	-	**0.0110**	**0.0023**	0.2188	0.0591	**0.0003**	0.2782
FFD	**0.0110**	-	0.5824	0.1733	0.4882	**<0.0001**	0.1321
BD	**0.0023**	0.5824	-	0.0581	0.2159	**<0.0001**	**0.0418**
RD	0.2188	0.1733	0.0581	-	0.4990	**<0.0001**	0.8825
LOV	0.0591	0.4882	0.2159	0.4990	-	**<0.0001**	0.4105
VEGAN	**0.0003**	**<0.0001**	**<0.0001**	**<0.0001**	**<0.0001**	**-**	**<0.0001**
PV	0.2782	0.1321	**0.0418**	0.8825	0.4105	**<0.0001**	-

$\bar{x}–\mathrm{average}\;\mathrm{value};\;$bold—statistically significant differences (*p* ≤ 0.05).

**Table 5:** The comparison of the average content of total fats, PUFA, and cholesterol between the diets (g/day; mg/day).

**Total Fats (g/day)**							
	**MFD**	**FFD**	**BD**	**RD**	**LOV**	**VEGAN**	**PV**
$\bar{x}$ ± SD	71.7 ± 3.5	75.1 ± 3.0	74.8 ± 3.1	86.8 ± 8.6	76.9 ± 4.1	70.7 ± 7.1	75.1 ± 2.6
MFD	-	0.1461	0.1723	**<0.0001**	**0.0248**	0.6478	0.1397
FFD	0.1461	-	0.9278	**<0.0001**	0.4108	0.0580	0.9809
BD	0.1723	0.9278	-	**<0.0001**	0.3616	0.0705	0.9087
RD	**<0.0001**	**<0.0001**	**<0.0001**	**-**	**<0.0001**	**<0.0001**	**<0.0001**
LOV	**0.0248**	0.4108	0.3616	**<0.0001**	**-**	**0.076**	0.4245
VEGAN	0.6478	0.0580	0.0705	**<0.0001**	**0.076**	-	0.0551
PV	0.1397	0.9809	0.9087	**<0.0001**	0.4245	0.0551	-
**Long-Chain Pufa Acids (g/day)**							
	**MFD**	**FFD**	**BD**	**RD**	**LOV**	**VEGAN**	**PV**
$\bar{x}$ ± SD	0.601 ± 1.1	0.032 ± 0.03	0.602 ± 1.1	0.106 ± 0.16	0.052 ± 0.04	0.004 ± 0.01	0.024 ± 0.03
MFD	**-**	**0.0368**	0.9977	0.0682	**0.0438**	**0.0289**	**0.0344**
FFD	**0.0368**	**-**	**0.0365**	0.7821	0.9397	0.9184	0.9772
BD	0.9977	**0.0365**	**-**	0.0678	**0.0435**	**0.0287**	**0.0342**
RD	0.0682	0.7821	0.0678	-	0.8407	0.7047	0.7602
LOV	**0.0438**	0.9397	**0.0435**	0.8407	-	0.8586	0.9169
VEGAN	**0.0289**	0.9184	**0.0287**	0.7047	0.8586	-	0.9411
PV	**0.0344**	0.9772	**0.0342**	0.7602	0.9169	0.9411	-
**Cholesterol (mg/day)**							
	**MFD**	**FFD**	**BD**	**RD**	**LOV**	**VEGAN**	**PV**
$\bar{x}$ ± SD	159.7 ± 98.8	202.4 ± 105.4	217.4 ± 101.3	369.9 ± 185	306.5 ± 150.9	0.1 ± 0.0	190.9 ± 95.2
MFD	-	0.4215	0.2777	**0.0002**	**0.0070**	**0.0036**	0.5562
FFD	0.4215	-	0.7760	**0.0036**	0.0525	**0.0003**	0.8285
BD	0.2777	0.7760	-	**0.0052**	0.0958	**<0.0001**	0.6165
RD	**0.0002**	**0.0036**	**0.0052**	-	0.2335	**<0.0001**	**0.0012**
LOV	**0.0070**	0.0525	0.0958	0.2335	-	**<0.0001**	**0.0319**
VEGAN	**0.0036**	**0.0003**	**<0.0001**	**<0.0001**	**<0.0001**	**-**	**0.0006**
PV	0.5562	0.8285	0.6165	**0.0012**	**0.0319**	**0.0006**	-

$\bar{x}–\mathrm{average}\;\mathrm{value};\;$SD—standard deviation; bold—statistically significant differences (*p* ≤ 0.05).

**Table 6:** The comparison of the average content of carbohydrates, dietary fiber, saccharose, lactose, and starch between the diets (g/day).

**Total Carbohydrates (g/day)**							
	**MFD**	**FFD**	**BD**	**RD**	**LOV**	**VEGAN**	**PV**
$\bar{x}$ ± SD	293.2 ± 16.54	290.6 ± 7.01	288.8 ± 7.58	257.9 ± 24.29	284.5 ± 13.19	304.7 ± 22.17	293 ± 9.04
MFD	-	0.7082	0.5285	**<0.0001**	0.2160	0.1059	0.9780
FFD	0.7082	-	0.7974	**<0.0001**	0.3855	**0.0480**	0.7287
BD	0.5285	0.7974	-	**<0.0001**	0.5401	**0.0264**	0.5466
RD	**<0.0001**	**<0.0001**	**<0.0001**	**-**	**0.0003**	**<0.0001**	**<0.0001**
LOV	0.2160	0.3855	0.5401	**0.0003**	-	**0.0053**	0.2262
VEGAN	0.1059	**0.0480**	**0.0264**	**<0.0001**	**0.0053**	**-**	0.1003
PV	0.9780	0.7287	0.5466	**<0.0001**	0.2262	0.1003	-
**Dietary Fiber (g/day)**							
	**MFD**	**FFD**	**BD**	**RD**	**LOV**	**VEGAN**	**PV**
$\bar{x}$ ± SD	41 ± 5.04	38 ± 5.05	38 ± 4.9	17 ± 3.97	37 ± 5.66	46 ± 7.77	38 ± 4.78
MFD	-	0.1621	0.1596	**<0.0001**	0.1147	0.0677	0.2029
FFD	0.1621	-	0.9934	**<0.0001**	0.8540	**0.0017**	0.8985
BD	0.1596	0.9934	-	**<0.0001**	0.8606	**0.0017**	0.8919
RD	**<0.0001**	**<0.0001**	**<0.0001**	-	**<0.0001**	**<0.0001**	**<0.0001**
LOV	0.1147	0.8540	0.8606	**<0.0001**	-	**0.0010**	0.7554
VEGAN	0.0677	**0.0017**	**0.0017**	**<0.0001**	**0.0010**	-	**0.0025**
PV	0.2029	0.8985	0.8919	**<0.0001**	0.7554	**0.0025**	-
**Saccharose (g/day)**							
	**MFD**	**FFD**	**BD**	**RD**	**LOV**	**VEGAN**	**PV**
$\bar{x}$ ± SD	22.97 ± 7.59	24.7 ± 7.69	24.7 ± 7.74	40 ± 13.73	22.86 ± 7.92	23.59 ± 8.11	24.5 ± 7.6
MFD	-	0.6637	0.6653	**<0.0001**	0.9780	0.8771	0.7016
FFD	0.6637	-	0.9982	**0.0003**	0.6438	0.7791	0.9587
BD	0.6653	0.9982	-	**0.0003**	0.6454	0.7808	0.9605
RD	**<0.0001**	**0.0003**	**0.0003**	**-**	**<0.0001**	**0.0001**	**0.0002**
LOV	0.9780	0.6438	0.6454	**<0.0001**	-	0.8554	0.6813
VEGAN	0.8771	0.7791	0.7808	**0.0001**	0.8554	-	0.8191
PV	0.7016	0.9587	0.9605	**0.0002**	0.6813	0.8191	-
**Lactose (g/day)**							
	**MFD**	**FFD**	**BD**	**RD**	**LOV**	**VEGAN**	**PV**
$\bar{x}$ ± SD	0.051 ± 0.088	12.4 ± 7.42	11.87 ± 7.27	4.41 ± 4.56	12.07 ± 6.72	0.049 ± 0.09	12.61 ± 7.48
MFD	-	**<0.0001**	**<0.0001**	0.0940	**<0.0001**	0.9994	**<0.0001**
FFD	**<0.0001**	-	0.8368	**0.0028**	0.9005	**<0.0001**	0.9349
BD	**<0.0001**	0.8368	-	**0.0050**	0.9355	**<0.0001**	0.7737
RD	0.0940	**0.0028**	**0.0050**	**-**	**0.0040**	0.0939	0.0022
LOV	**<0.0001**	0.9005	0.9355	**0.0040**	**-**	**<0.0001**	0.8363
VEGAN	0.9994	**<0.0001**	**<0.0001**	0.0939	**<0.0001**	**-**	**<0.0001**
PV	**<0.0001**	0.9349	0.7737	**0.0022**	0.8363	**<0.0001**	-
**Starch (g/day)**							
	**MFD**	**FFD**	**BD**	**RD**	**LOV**	**VEGAN**	**PV**
$\bar{x}$ ± SD	157.7 ± 27.8	147.8 ± 31.3	147.0 ± 29.3	166.9 ± 17.4	145.6 ± 30.3	161.3 ± 20.3	149.6 ± 33.3
MFD	-	0.4250	0.3896	0.4573	0.3329	0.7714	0.5149
FFD	0.4250	-	0.9496	0.1259	0.8633	0.2778	0.8828
BD	0.3896	0.9496	-	0.1115	0.9132	0.2512	0.8333
RD	0.4573	0.1259	0.1115	-	0.0897	0.6499	0.1656
LOV	0.3329	0.8633	0.9132	0.0897	-	0.2096	0.7494
VEGAN	0.7714	0.2778	0.2512	0.6499	0.2096	-	0.3474
PV	0.5149	0.8828	0.8333	0.1656	0.7494	0.3474	-

$\bar{x}–\mathrm{average}\;\mathrm{value};\;$SD—standard deviation; bold—statistically significant differences (*p* ≤ 0.05).

**Table 7:** The average content of minerals in the analyzed diets.

Mineral	$\mathrm{MFD}\;\bar{x}$ **± SD**	$\mathrm{FFD}\;\bar{x}$ **± SD**	$\mathrm{BD}\;\bar{x}$ **± SD**	$\mathrm{RD}\;\bar{x}$ **± SD**	$\mathrm{LOV}\;\bar{x}$ **± SD**	$\mathrm{VEGAN}\;\bar{x}$ **± SD**	$\mathrm{PV}\;\bar{x}$ **± SD**
Sodium (mg)	1175 ± 519.1	1501 ± 575.1	1414 ± 496.1	3527 ± 813	1499 ± 408.4	915 ± 290.5	1490 ± 481
Potassium (mg)	4324.2 ± 743	4242.3 ± 583	4390 ± 616	2594.7 ± 594	4037.5 ± 63	4378.4 ± 845.5	4052 ± 473
Calcium (mg)	452.4 ± 73.8	1281.7 ± 239.3	1169.1 ± 310.2	444.1 ± 157.7	1363.1 ± 267	610.2 ± 107.4	1360.7 ± 299.2
Phosphorus (mg)	1509.5 ± 121.4	1704.8 ± 184.5	1698 ± 189.7	1099.6 ± 178.9	1674.4 ± 118.1	1449.7 ± 145.2	1674.4 ± 193.7
Magnesium (mg)	558.6 ± 66.1	505.3 ± 60.7	508.7 ± 65.2	261.1 ± 70.2	484.8 ± 71.6	589.3 ± 49.1	498.5 ± 72.2
Iron (mg)	18.2 ± 2.2	15.3 ± 1.5	15.4 ± 1.3	11.2 ± 2.2	15.7 ± 1.5	19.8 ± 1.9	15.3 ± 2
Zinc (mg)	12.1 ± 1.9	12.9 ± 1.6	12.6 ± 1.5	10.6 ± 1.8	12.9 ± 1.4	12.6 ± 1.2	12.8 ± 1.6
Copper (mg)	2.4 ± 0.3	2.1 ± 0.3	2.1 ± 0.3	1.0 ± 0.2	2.1 ± 04	2.7 ± 0.4	2.2 ± 0.3
Manganese (mg)	8.0 ± 1.8	7.2 ± 1.7	7.0 ± 1.6	3.0 ± 1.1	7.1 ± 1.3	9.0 ± 1.5	7.2 ± 1.6
Iodine (µg)	62.8 ± 46.4	46.9 ± 20.3	57.9 ± 19.1	87.2 ± 45.0	37.6 ± 9.2	29.4 ± 10.3	39.2 ± 11.1

$\bar{x}—\mathrm{average}\;\mathrm{value};\;$SD—standard deviation.

**Table 8:** Comparison of the average content of vitamins in the analyzed diets.

Vitamin	$\mathrm{MFD}\;\bar{x}$ **± SD**	$\mathrm{FFD}\;\bar{x}$ **± SD**	$\mathrm{BD}\;\bar{x}$ **± SD**	$\mathrm{RD}\;\bar{x}$ **± SD**	$\mathrm{LOV}\;\bar{x}$ **± SD**	$\mathrm{VEGAN}\;\bar{x}$ **± SD**	$\mathrm{PV}\;\bar{x}$ **± SD**
Vitamin A (µg)	1573.8 ± 682.9	1697.8 ± 775.6	1727.1 ± 739.7	1000 ± 491.6	1769.5 ± 766.3	1473.6 ± 709.2	1665.4 ± 641.3
Retinol (µg)	126.1 ± 96.3	243.5 ± 87.9	242.1 ± 83.6	682.7 ± 435.5	352.7 ± 131.3	22.7 ± 31.9	286.8 ± 68.8
β-carotene (µg)	8463.2 ± 4300.5	8757.8 ± 4519	8942.3 ± 4514.3	1917.6 ± 2169.6	8533.6 ± 4049.1	8462.3 ± 4297	8283.2 ± 3901
Vitamin E (mg)	19.4 ± 3.8	15.3 ± 3.4	15.7 ± 3.5	9.6 ± 4.8	15.5 ± 3.2	18.7 ± 3.4	15.1 ± 3.5
Thiamin (mg)	1.9 ± 0.3	1.7 ± 0.4	1.7 ± 0.4	1.5 ± 0.4	1.5 ± 0.3	2 ± 0.4	1.6 ± 0.3
Riboflavin (mg)	1.6 ± 0.2	1.9 ± 0.4	1.9 ± 0.4	1.4 ± 0.3	2.0 ± 0.4	1.4 ± 0.3	2 ± 0.3
Niacin (mg)	21.7 ± 5.6	17.6 ± 4.6	10.1 ± 5.2	16 ± 4.4	13.6 ± 3.6	15.6 ± 3.3	13.6 ± 3.0
Vitamin B_6_ (mg)	3.0 ± 0.5	2.7 ± 0.5	2.9 ± 0.6	1.7 ± 0.5	2.5 ± 0.4	2.7 ± 0.5	2.4 ± 0.4
Vitamin C (mg)	208.3 ± 52.3	216.8 ± 55.3	219.9 ± 56.9	70.9 ± 47	227.9 ± 69	210.1 ± 53.9	210.6 ± 46.8
Folates (µg)	496.4 ± 61.3	485.4 ± 71.2	490.6 ± 74.2	216.5 ± 57.6	507.9 ± 78.1	520.3 ± 87.8	494.6 ± 65.1
Vitamin B_12_ (µg)	4.1 ± 1.8	2.7 ± 0.8	3.5 ± 1.3	4.1 ± 2.1	3.1 ± 0.9	2.2 ± 1.3	2.6 ± 0.8
Vitamin D (µg)	4.3 ± 4.7	1.0 ± 0.8	3.4 ± 4.3	3.5 ± 2.3	1.5 ± 0.7	1.0 ± 0.8	1.0 ± 0.7

$\bar{x}$—average value, SD—standard deviation.

## Supplementary Materials

The following are available online at https://www.mdpi.com/2072-6643/12/10/2986/s1, Table S1: Differences in mineral content, Table S2: The presence of statistically significant differences in the content of vitamins between the diets.

## Abbreviations

BD	The basic diet
CKD	chronic kidney disease
CVD	cardiovascular disease
DHA	Docosahexaenoic Acid
EPA	Eicosapentaenoic acid
FFD	Diet that eliminates fish, seafood and shellfish
FOODMAP	fermentable oligosaccharides, disaccharides, monosaccharides and polyols
LDL	Low-density lipoprotein
LOV	The lacto-ovo-vegetarian diet
MFD	The milk-free diet
MTHFR	methylenetetrahydrofolate reductase
MUFA	monounsaturated fatty acids
PUFA	polyunsaturated fatty acids
RD	regular diet of Poles
SFA	saturated fatty acids
VEGAN	The vegan diet
PV	The modification of a vegetarian diet with fish
