# Supplementary material for: The Change in the Content of Nutrients in Diets Eliminating Products of Animal Origin in Comparison to a Regular Diet from the Area of Middle-Eastern Europe
Source: Nutrients. 2020 Sep 29;12(10):2986. doi: 10.3390/nu12102986 (PMC7599827; doi:10.3390/nu12102986)
Supplement: Supplementary file 1 [file nutrients-12-02986-s001.pdf]

Table S1. Differences in mineral content

**SODIUM**	MFD	FFD	BD	RD	LOV	VEGAN	PV
MFD	-	0.1758	0.3203	**<0.0001**	0.1795	0.2795	0.1918
FFD	0.1758	-	0.7145	**<0.0001**	0.9907	**0.0167**	0.9604
BD	0.3203	0.7145	-	**<0.0001**	0.7232	**0.0404**	0.7518
RD	**<0.0001**	**<0.0001**	**<0.0001**	-	**<0.0001**	**<0.0001**	**<0.0001**
LOV	0.1795	0.9907	0.7232	**<0.0001**	-	**0.0172**	0.9698
VEGAN	0.2795	**0.0167**	**0.0404**	**<0.0001**	**0.0172**	-	**0.0189**
PV	0.1918	0.9604	0.7518	**<0.0001**	0.9698	**0.0189**	-
**POTASSIUM**	MFD	FFD	BD	RD	LOV	VEGAN	PV
MFD	-	0.7795	0.8219	**<0.0001**	0.3288	0.8530	0.3546
FFD	0.7795	-	0.6138	**<0.0001**	0.4846	0.6420	0.5171
BD	0.8219	0.6138	-	**<0.0001**	0.2307	0.9682	0.2509
RD	**<0.0001**	**<0.0001**	**<0.0001**	-	**<0.0001**	**<0.0001**	**<0.0001**
LOV	0.3288	0.4846	0.2307	**<0.0001**	-	0.2463	0.9591
VEGAN	0.8530	0.6420	0.9682	**<0.0001**	0.2463	-	0.2675
PV	0.3546	0.5171	0.2509	**<0.0001**	0.9591	0.2675	-
**CALCIUM**	MFD	FFD	BD	RD	LOV	VEGAN	PV
MFD	-	**<0.0001**	**<0.0001**	0.9349	**<0.0001**	0.1227	**<0.0001**
FFD	**<0.0001**	-	0.2687	**<0.0001**	0.4228	**<0.0001**	0.4361
BD	**<0.0001**	0.2687	-	**<0.0001**	0.0590	**<0.0001**	0.0621
RD	0.9349	**<0.0001**	**<0.0001**	-	**<0.0001**	0.1046	**<0.0001**
LOV	**<0.0001**	0.4228	0.0590	**<0.0001**	-	**<0.0001**	0.9816
VEGAN	0.1227	**<0.0001**	**<0.0001**	0.1046	**<0.0001**	-	**<0.0001**
PV	**<0.0001**	0.4361	0.0621	**<0.0001**	0.9816	**<0.0001**	-
**PHOSPHORUS**	MFD	FFD	BD	RD	LOV	VEGAN	PV
MFD	-	**0.0100**	**0.0128**	**<0.0001**	**0.0284**	0.4192	**0.0284**
FFD	**0.0100**	-	0.9273	**<0.0001**	0.6814	**0.0009**	0.6814
BD	**0.0128**	0.9273	-	**<0.0001**	0.7494	**0.0013**	0.7494
RD	**<0.0001**	**<0.0001**	**<0.0001**	-	**<0.0001**	**<0.0001**	**<0.0001**
LOV	**0.0284**	0.6814	0.7494	**<0.0001**	-	**0.0033**	>0.9999
VEGAN	0.4192	**0.0009**	**0.0013**	**<0.0001**	**0.0033**	-	**0.0033**
PV	**0.0284**	0.6814	0.7494	**<0.0001**	>0.9999	**0.0033**	-
**MAGNESIUM**	MFD	FFD	BD	RD	LOV	VEGAN	PV
MFD	-	0.0734	0.0937	**<0.0001**	**0.0143**	0.2977	**0.0444**
FFD	0.0734	-	0.9061	**<0.0001**	0.4880	**0.0056**	0.8187
BD	0.0937	0.9061	-	**<0.0001**	0.4175	**0.0077**	0.7285
RD	**<0.0001**	**<0.0001**	**<0.0001**	-	**<0.0001**	**<0.0001**	**<0.0001**
LOV	**0.0143**	0.4880	0.4175	**<0.0001**	-	**0.0007**	0.6418
VEGAN	0.2977	**0.0056**	**0.0077**	**<0.0001**	**0.0007**	-	**0.0029**
PV	**0.0444**	0.8187	0.7285	**<0.0001**	0.6418	**0.0029**	-
**IRON**	MFD	FFD	BD	RD	LOV	VEGAN	PV
MFD	-	**0.0009**	**0.0014**	**<0.0001**	**0.0044**	**0.0484**	**0.0009**
FFD	**0.0009**	-	0.8963	**<0.0001**	0.6043	**<0.0001**	0.9778
BD	**0.0014**	0.8963	-	**<0.0001**	0.6979	**<0.0001**	0.8743
RD	**<0.0001**	**<0.0001**	**<0.0001**	-	**<0.0001**	**<0.0001**	**<0.0001**
LOV	**0.0044**	0.6043	0.6979	**<0.0001**	-	**<0.0001**	0.5851
VEGAN	**0.0484**	**<0.0001**	**<0.0001**	**<0.0001**	**<0.0001**	-	**<0.0001**

PV	**0.0009**	0.9778	0.8743	**<0.0001**	0.5851	**<0.0001**	-
**ZINC**	MFD	FFD	BD	RD	LOV	VEGAN	PV
MFD	-	0.2353	0.4921	**0.0357**	0.2599	0.5072	0.3442
FFD	0.2353	-	0.6136	**0.0014**	0.9512	0.5971	0.8070
BD	0.4921	0.6136	-	**0.0061**	0.6572	0.9810	0.7942
RD	**0.0357**	**0.0014**	**0.0061**	**-**	**0.0017**	**0.0065**	**0.0029**
LOV	0.2599	0.9512	0.6572	**0.0017**	-	0.6401	0.8546
VEGAN	0.5072	0.5971	0.9810	**0.0065**	0.6401	-	0.7759
PV	0.3442	0.8070	0.7942	**0.0029**	0.8546	0.7759	-
**COPPER**	MFD	FFD	BD	RD	LOV	VEGAN	PV
MFD	-	**0.0466**	**0.0383**	**<0.0001**	**0.0359**	0.0766	0.0738
FFD	**0.0466**	-	0.9314	**<0.0001**	0.9090	**0.0003**	0.8330
BD	**0.0383**	0.9314	-	**<0.0001**	0.9775	**0.0002**	0.7666
RD	**<0.0001**	**<0.0001**	**<0.0001**	**-**	**<0.0001**	**<0.0001**	**<0.0001**
LOV	**0.0359**	0.9090	0.9775	**<0.0001**	-	**0.0002**	0.7452
VEGAN	0.0766	**0.0003**	**0.0002**	**<0.0001**	**0.0002**	**-**	**0.0006**
PV	0.0738	0.8330	0.7666	**<0.0001**	0.7452	**0.0006**	-
**MANGANESE**	MFD	FFD	BD	RD	LOV	VEGAN	PV
MFD	-	0.2735	0.1858	**<0.0001**	0.2128	0.1402	0.2799
FFD	0.2735	-	0.8166	**<0.0001**	0.8780	**0.0116**	0.9882
BD	0.1858	0.8166	-	**<0.0001**	0.9374	**0.0062**	0.8051
RD	**<0.0001**	**<0.0001**	**<0.0001**	**-**	**<0.0001**	**<0.0001**	**<0.0001**
LOV	0.2128	0.8780	0.9374	**<0.0001**	-	**0.0077**	0.8664
VEGAN	0.1402	**0.0116**	**0.0062**	**<0.0001**	**0.0077**	**-**	**0.0121**
PV	0.2799	0.9882	0.8051	**<0.0001**	0.8664	**0.0121**	-
**IODINE**	MFD	FFD	BD	RD	LOV	VEGAN	PV
MFD	-	0.1976	0.6870	0.0510	**0.0440**	**0.0083**	0.0583
FFD	0.1976	-	0.3729	**0.0016**	0.4543	0.1590	0.5333
BD	0.6870	0.3729	-	**0.0197**	0.1038	**0.0234**	0.1326
RD	0.0510	**0.0016**	**0.0197**	**-**	**0.0001**	**<0.0001**	**0.0002**
LOV	**0.0440**	0.4543	0.1038	**0.0001**	-	0.5038	0.8997
VEGAN	**0.0083**	0.1590	**0.0234**	**<0.0001**	0.5038	-	0.4274
PV	0.0583	0.5333	0.1326	**0.0002**	0.8997	0.4274	-

Table S2. The presence of statistically significant differences in the content of vitamins between the diets

**VIT.A**	MFD	FFD	BD	RD	LOV	VEGAN	PV
MFD	-	0.6902	0.6224	0.0687	0.5298	0.7474	0.7684
FFD	0.6902	-	0.9250	**0.0278**	0.8178	0.4718	0.9170
BD	0.6224	0.9250	-	**0.0221**	0.8916	0.4162	0.8482
RD	0.0687	**0.0278**	**0.0221**	**-**	**0.0157**	0.1313	**0.0356**
LOV	0.5298	0.8178	0.8916	**0.0157**	-	0.3431	0.7380
VEGAN	0.7474	0.4718	0.4162	0.1313	0.3431	-	0.5380
PV	0.7684	0.9170	0.8482	**0.0356**	0.7380	0.5380	-
**RETINOL**	MFD	FFD	BD	RD	LOV	VEGAN	PV
MFD	-	0.1586	0.1634	**<0.0001**	**0.0077**	0.2129	0.0552
FFD	0.1586	-	0.9867	**<0.0001**	0.1888	**0.0093**	0.6005

BD	0.1634	0.9867	-	**<0.0001**	0.1834	**0.0097**	0.5890
RD	**<0.0001**	**<0.0001**	**<0.0001**	**-**	**0.0002**	**<0.0001**	**<0.0001**
LOV	**0.0077**	0.1888	0.1834	**0.0002**	-	0.0002	0.4255
VEGAN	0.2129	**0.0093**	**0.0097**	**<0.0001**	0.0002	**-**	**0.0021**
PV	0.0552	0.6005	0.5890	**<0.0001**	0.4255	**0.0021**	-
**BETA-CAROTENE**	MFD	FFD	BD	RD	LOV	VEGAN	PV
MFD	-	0.8709	0.7916	**0.0006**	0.9690	0.9996	0.9290
FFD	0.8709	-	0.9189	**0.0003**	0.9016	0.8705	0.7935
BD	0.7916	0.9189	-	**0.0002**	0.8217	0.7912	0.7163
RD	**0.0006**	**0.0003**	**0.0002**	**-**	**0.0005**	**0.0006**	**0.0008**
LOV	0.9690	0.9016	0.8217	**0.0005**	-	0.9686	0.8901
VEGAN	0.9996	0.8705	0.7912	**0.0006**	0.9686	-	0.9213
PV	0.9290	0.7935	0.7163	**0.0008**	0.8901	0.9213	-
**VIT.E**	MFD	FFD	BD	RD	LOV	VEGAN	PV
MFD	-	**0.0158**	**0.0292**	**<0.0001**	**0.0225**	0.6782	**0.0117**
FFD	**0.0158**	-	0.8045	**0.0011**	0.8882	**0.0431**	0.9089
BD	**0.0292**	0.8045	-	**0.0005**	0.9147	0.0742	0.7175
RD	**<0.0001**	**0.0011**	**0.0005**	**-**	**0.0007**	**<0.0001**	**0.0016**
LOV	**0.0225**	0.8882	0.9147	**0.0007**	-	0.0590	0.7988
VEGAN	0.6782	**0.0431**	0.0742	**<0.0001**	0.0590	-	**0.0331**
PV	**0.0117**	0.9089	0.7175	**0.0016**	0.7988	**0.0331**	-
**THIAMIN**	MFD	FFD	BD	RD	LOV	VEGAN	PV
MFD	-	0.1775	0.2413	**0.0470**	**0.0485**	0.5150	0.0650
FFD	0.1775	-	0.8570	0.5105	0.5193	**0.0478**	0.6090
BD	0.2413	0.8570	-	0.4025	0.4102	0.0708	0.4896
RD	**0.0470**	0.5105	0.4025	-	0.9891	**0.0094**	0.8830
LOV	**0.0485**	0.5193	0.4102	0.9891	-	**0.0097**	0.8938
VEGAN	0.5150	**0.0478**	0.0708	**0.0094**	**0.0097**	**-**	**0.0138**
PV	0.0650	0.6090	0.4896	0.8830	0.8938	**0.0138**	-
**RIBOFLAVIN**	MFD	FFD	BD	RD	LOV	VEGAN	PV
MFD	**-**	**0.0133**	**0.0247**	0.2294	**0.0025**	0.3320	**0.0098**
FFD	**0.0133**	-	0.8071	**0.0004**	0.5447	**0.0008**	0.9062
BD	**0.0247**	0.8071	-	**0.0008**	0.3963	**0.0017**	0.7175
RD	0.2294	**0.0004**	**0.0008**	**-**	**<0.0001**	0.8142	**0.0003**
LOV	**0.0025**	0.5447	0.3963	**<0.0001**	**-**	**0.0001**	0.6254
VEGAN	0.3320	**0.0008**	**0.0017**	0.8142	**0.0001**	-	**0.0005**
PV	**0.0098**	0.9062	0.7175	**0.0003**	0.6254	**0.0005**	-
**NIACIN**	MFD	FFD	BD	RD	LOV	VEGAN	PV
MFD	**-**	**0.0419**	0.4278	**0.0047**	**<0.0001**	**0.0028**	**0.0001**
FFD	**0.0419**	**-**	0.2057	0.3969	**0.0403**	0.3056	**0.0434**
BD	0.4278	0.2057	-	**0.0369**	**0.0013**	**0.0241**	**0.0014**
RD	**0.0047**	0.3969	**0.0369**	-	0.2191	0.8579	0.2314
LOV	**<0.0001**	**0.0403**	**0.0013**	0.2191	-	0.2925	0.9739
VEGAN	**0.0028**	0.3056	**0.0241**	0.8579	0.2925	-	0.3076
PV	**0.0001**	**0.0434**	**0.0014**	0.2314	0.9739	0.3076	-
**VIT.B_6**	MFD	FFD	BD	RD	LACTO	VEGAN	PV

MFD	-	0.1275	0.6135	**<0.0001**	**0.0181**	0.1532	**0.0120**
FFD	0.1275	-	0.3037	**<0.0001**	0.3811	0.9216	0.3007
BD	0.6135	0.3037	-	**<0.0001**	0.0595	0.3518	**0.0416**
RD	**<0.0001**	**<0.0001**	**<0.0001**	**-**	**0.0005**	**<0.0001**	**0.0009**
LOV	0.0181	0.3811	0.0595	**0.0005**	-	0.3304	0.8723
VEGAN	0.1532	0.9216	0.3518	**<0.0001**	0.3304	-	0.2577
PV	**0.0120**	0.3007	**0.0416**	**0.0009**	0.8723	0.2577	-
**VIT.C**	MFD	FFD	BD	RD	LOV	VEGAN	PV
MFD	-	0.7327	0.6378	**<0.0001**	0.4287	0.9416	0.9266
FFD	0.7327	-	0.8970	**<0.0001**	0.6518	0.7884	0.8029
BD	0.6378	0.8970	-	**<0.0001**	0.7474	0.6909	0.7048
RD	**<0.0001**	**<0.0001**	**<0.0001**	**-**	**<0.0001**	**<0.0001**	**<0.0001**
LOV	0.4287	0.6518	0.7474	**<0.0001**	-	0.4724	0.4840
VEGAN	0.9416	0.7884	0.6909	**<0.0001**	0.4724	-	0.9850
PV	0.9266	0.8029	0.7048	**<0.0001**	0.4840	0.9850	-
**FOLATES**	MFD	FFD	BD	RD	LOV	VEGAN	PV
MFD	-	0.7323	0.8574	**<0.0001**	0.7192	0.4556	0.9555
FFD	0.7323	-	0.8710	**<0.0001**	0.4836	0.2780	0.7747
BD	0.8574	0.8710	-	**<0.0001**	0.5900	0.3553	0.9014
RD	**<0.0001**	**<0.0001**	**<0.0001**	**-**	**<0.0001**	**<0.0001**	**<0.0001**
LOV	0.7192	0.4836	0.5900	**<0.0001**	-	0.6981	0.6779
VEGAN	0.4556	0.2780	0.3553	**<0.0001**	0.6981	-	0.4228
PV	0.9555	0.7747	0.9014	**<0.0001**	0.6779	0.4228	-
**VIT.B$_{12}$**	MFD	FFD	BD	RD	LOV	VEGAN	PV
MFD	**-**	**0.0259**	0.3019	0.9989	0.0884	**0.0032**	**0.0173**
FFD	**0.0259**	**-**	0.2191	**0.0260**	0.5834	0.4371	0.8721
BD	0.3019	0.2191	-	0.3026	0.4927	**0.0473**	0.1655
RD	0.9989	**0.0260**	0.3026	-	0.0886	**0.0032**	**0.0174**
LOV	0.0884	0.5834	0.4927	0.0886	-	0.1872	0.4785
VEGAN	**0.0032**	0.4371	**0.0473**	**0.0032**	0.1872	-	0.5372
PV	**0.0173**	0.8721	0.1655	**0.0174**	0.4785	0.5372	-
**VIT.D**	MFD	FFD	BD	RD	LOV	VEGAN	PV
MFD	-	**0.0072**	0.4768	0.5363	**0.0193**	**0.0075**	**0.0066**
FFD	**0.0072**	**-**	**0.0435**	**0.0351**	0.7093	0.9912	0.9737
BD	0.4768	**0.0435**	-	0.9255	0.0968	**0.0446**	**0.0404**
RD	0.5363	**0.0351**	0.9255	-	0.0800	**0.0360**	**0.0325**
LOV	**0.0193**	0.7093	0.0968	0.0800	-	0.7175	0.6849
VEGAN	**0.0075**	0.9912	**0.0446**	**0.0360**	0.7175	-	0.9649
PV	**0.0066**	0.9737	**0.0404**	**0.0325**	0.6849	0.9649	-

bold– statistically significant differences
